# Dispersion Stability and Tribological Properties of Cold Plasma-Modified h-BN Nanofluid

**DOI:** 10.3390/nano15110874

**Published:** 2025-06-05

**Authors:** Zhenjing Duan, Ziheng Wang, Yishuai Jia, Shuaishuai Wang, Peng Bian, Ji Tan, Jinlong Song, Xin Liu

**Affiliations:** State Key Laboratory of High-Performance Precision Manufacturing, Dalian University of Technology, Dalian 116024, China

**Keywords:** h-BN nanoparticles, nanofluid, cold plasma, coefficient of friction, surface roughness

## Abstract

h-BN spherical nanoparticles, known as white graphene, have good anti-wear properties, long service life, chemical inertness, and stability, which provide superior lubricating performance as a solid additive item to nanofluids. However, the poor dispersion stability of h-BN nanoparticles in nanofluids is a bottleneck that restricts their application. Currently, to prepare h-BN nanofluids with good dispersion stability, a cold plasma (CP) modification of h-BN nanoparticles is proposed in this study. In this research, h-BN nanofluid with added surfactant (SNL), CP-modified h-BN nanofluid with N_2_ as the working gas (CP(N_2_)NL), and CP-modified h-BN nanofluid with O_2_ as the working gas (CP(O_2_)NL) were prepared, separately. The mechanism of the dispersion stability of CP-modified h-BN nanofluid was analyzed using X-ray photoelectron spectroscopy (XPS), and the performance of CP-modified nanofluid was analyzed based on static observation of nanofluid, kinematic viscosity, and heat transfer properties. Finally, friction and wear experiments were conducted to further analyze the tribological performance of h-BN nanofluids based on the coefficient of friction, 3D surface morphology, surface roughness (Sa), scratches, and micro-morphology. The results show that CP-modified h-BN nanofluid has excellent dispersed suspension stability and can be statically placed for more than 336 h. The CP-modified h-BN nanofluid showed stable friction-reducing, anti-wear, and heat transfer performance, in which the coefficient of friction of h-BN nanofluid was about 0.66 before and after 24 h of settling. The Sa value of the sample was reduced by 31.6–49.2% in comparison with pure cottonseed oil (CO).

## 1. Introduction

Nanofluid is a new kind of composite material formed by uniformly dispersing nano-scale solid nanoparticles in base fluid and is widely used in the lubrication field and heat transfer field [1,2,3,4]. In the field of mechanical transmission, such as gear transmission, chain transmission, and other mechanical transmission systems, nanofluids can fill the small gap between transmission components, forming a uniform lubrication film, improving transmission efficiency, and reducing energy loss [5,6]. Among the commonly used nanoparticles are metal nanoparticles (Cu and Ag nanoparticles), oxide nanoparticles (Al_2_O_3_, TiO_2_, SiO_2_, and CuO), sulfide nanoparticles (MoS_2_ and WS_2_), carbon nanomaterials (carbon nanotubes and diamond nanoparticles), nitrides (h-BN), and carbons (SiC) [7,8,9,10,11,12,13]. h-BN is commonly known as “white graphite” or “white graphene” because of its layer structure and crystal structure, which is similar to that of graphite [14,15]. h-BN spherical nanoparticles have good anti-wear properties, long service life, and stability, including high-temperature stability, as lubricant additives [16,17,18]. The preparation of nanofluids is a key step in research and applications.

Currently, the most commonly used preparation methods are physical methods, i.e., ultrasonic vibration and stirring; in addition, chemical methods, i.e., the addition of surfactant, are used to enhance the dispersion stability of nanoparticles based on physical methods [19,20]. Xia et al. employed a two-step method to prepare deionized water Al_2_O_3_ nanofluids with various surfactant mass fractions and found that polyvinylpyrrolidone (PVP) performed better in terms of dispersion and stability [21]. Altun et al. used Tween 80 and Tergitol NP-10 surfactants to prepare aqueous Al_2_O_3_ nanofluids and showed that the Tween 80 surfactant increased the thermal conductivity by 7.5% [22]. Mostafizur et al. compared the influence of various surfactants on the dispersion stability of methanol-based Al_2_O_3_ nanofluids and showed that cetyltrimethylammonium bromide (CTAB) was effective in improving the dispersion stability of Al_2_O_3_ nanofluid [23]. To prepare stable water-based MWCNT nanofluids, Sezer et al. used surfactants, oxidizing acid modification, and metal decoration, separately, and their study showed that surfactant and oxidizing acid treatments were two effective methods to enhance the stability and heat conductivity of MWCNT nanofluid dispersions [24]. Almanassra et al. used various surfactants to prepare aqueous carbon nanotube nanofluids and showed that GA and PVP were superior in terms of dispersion stability [25]. Ebrahim et al. employed surfactants such as GA and SDS to prepare aqueous graphene nanofluids and showed that both GA and SDS exhibited excellent dispersion stability [26]. Ibrahim et al. used ethanol and sodium deoxycholate (SDOC) surfactants to prepare aqueous GNP nanofluids with enhanced wettability, thermal conductivity, and tribological properties [27]. The above are water-based nanofluids prepared using different surfactants. For oil-based nanofluids, Gao et al. employed various surfactants to prepare CNT nanofluids and showed that APE-10 has excellent dispersion stability based on tribological analysis and nanofluid dispersion stabilization [28]. Musavi et al. used surfactant-containing nanofluids, surfactant-free nanofluids, and conventional cutting fluids to assist in turning of high-temperature alloys and found that the nanofluids prepared with the surfactant sodium dodecyl sulfate (SDS) had superior cutting performance [29]. Şirin et al. employed SDS and GA as surfactants to prepare hybrid hBN/GNP nanofluids, respectively, and found that SDS had better dispersion properties [30]. Wang et al. found that hybrid TiO_2_/CuO nanofluids prepared with sodium dodecyl sulfate (SLS) and CTAB have superior thermal conductivity compared to QF-DTk and PVP [31]. In addition, the researchers prepared ZnO nanofluids using a blend of surfactants (SDS/CTAB) and showed that the blend of surfactants kept the nanofluids dispersed and stable for 7 days [32]. Furthermore, researchers used stearic acid to modify TiO_2_ nanoparticles to enhance the dispersion stability of the nanofluids [33]. From the above studies, the dispersion stability of nanofluids is a major challenge in their application. Therefore, researchers have performed many studies on the preparation of dispersion-stable nanofluids, but most of them used chemical methods such as the addition of surfactants and acid modification. Further explorations are needed for methods that avoid the use of chemical reagents.

CP consists of high amounts of highly reactive particles (electrons, ions, free radicals, excited state atoms, molecules, etc.), which can be generated by bare electrode discharge, dielectric blocking discharge, or the use of microwave-frequency electromagnetic waves to stimulate gas discharge, i.e., microwave plasma (MP) [34,35]. CP has demonstrated excellent performance in assisting cutting, improving surface wetting, and material removal rates [36,37,38]. CP-treated materials can introduce new chemical groups into the surface layer of the material, which increases the surface energy of the material, and thus increases the wettability of the material [38,39,40]. Researchers dispersed CP-modified polymer powder in water and achieved excellent dispersion stability [41]. Studies on the feasibility of CP modification of h-BN nanoparticles to enhance the wettability of base oil, which in turn can help to prepare suspension-stabilized h-BN nanofluid, have not been performed.

In conclusion, researchers have conducted numerous studies to prepare nanofluids with better dispersion stability. Nevertheless, chemical methods (surfactant and acid modification) are mainly used to improve the poor dispersion stability. CP, as an eco-friendly modification, has been applied to the surface wettability modification of workpieces, and little research has been done on CP modification of h-BN nanoparticles for greater dispersion in the base fluid. In the present work, to prepare h-BN nanofluids with better dispersion stability, a vacuum apparatus for microwave plasma treatment of powders was built, and the mechanisms of modification were analyzed using XPS. Surfactant-containing alumina nanofluids, CP (N_2_)-modified h-BN nanofluids, and CP (O_2_)-modified h-BN nanofluids were prepared, and the physical properties of the various h-BN nanofluids were analyzed based on the kinematic viscosity, stability, and thermal diffusion coefficient of the h-BN nanofluids. Finally, friction wear tests (coefficient of friction, surface roughness Sa, micro-morphology, and scratch depth) were conducted to further prove the feasibility of using microwave plasma-modified h-BN nanoparticles to prepare uniformly suspended and stable nanofluids.

## 2. Experimental Details

### 2.1. Experimental Setup

**Figure 1:**
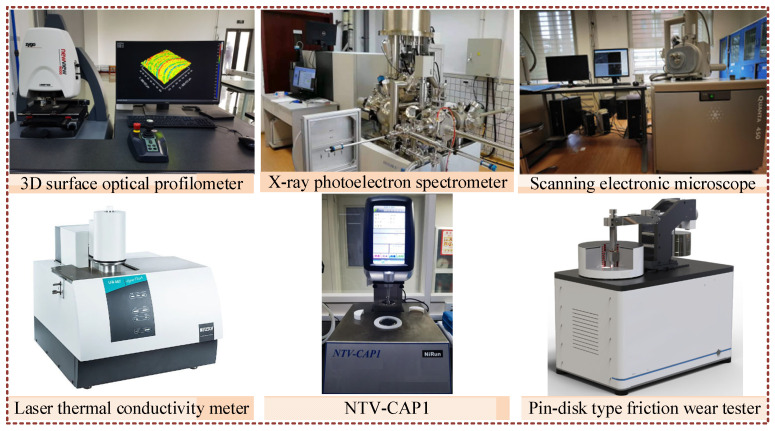
Experimental and analytical equipment.

As illustrated in Figure 1, friction and wear tests were carried out on a pin-on-disc friction wear tester (MPT20, Jinan xinbiao automation equipment Co., Ltd., Jinan, China). XPS (ESCALAB XI+, Thermo Scientific, Swindon, UK) was used to test workpiece surface composition. Microscopy (Zygo-9000, ZYGO Inc., Middlefield, CT, USA) was used to measure Sa. Micro-morphology was viewed using tungsten scanning electron microscopy (QUANTA 450, FEI Inc., Hillsboro, OR, USA). Kinematic viscosity was tested using a cone plate temperature-controlled rheometer (NTV-CAP1, NiRun, Shanghai, China). Nanofluid preparation was achieved using an ultrasonic vibration device (KQ5200E, Kunshan Ultrasonic Instrument Co., Kunshan, China) whose frequency was 40 kHz.

### 2.2. Material

**Figure 2:**
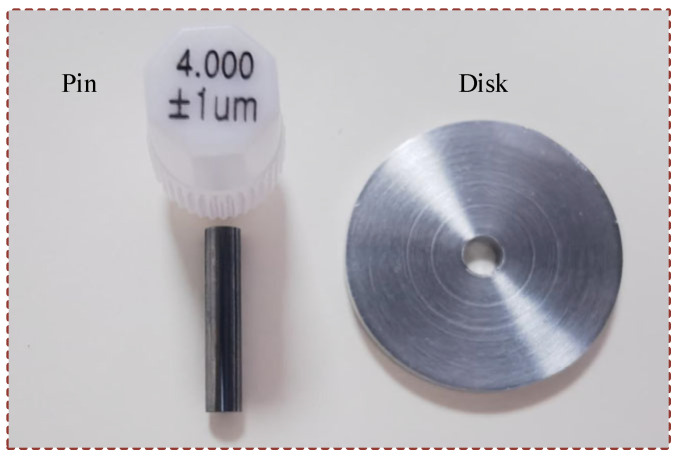
Pin and disk.

The friction discs were aluminum alloy 6061-T651 and the friction pins were tungsten steel, with a length of 20 mm, a diameter of 4 mm, and a chamfer of 0.2 mm (Figure 2). The materials used for the preparation of the nanofluid include h-BN nanoparticles (50 nm) and biodegradable CO. Table 1 shows the chemical composition of aluminum alloy 6061-T651.

### 2.3. Experimental Steps

#### 2.3.1. Cold Plasma-Modified h-BN Nanoparticles

**Figure 3:**
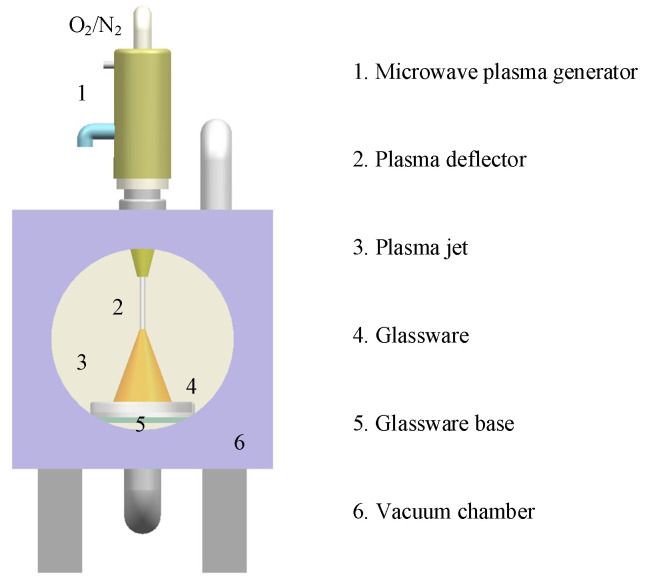
Microwave plasma equipment.

The modification of h-BN nanoparticles was carried out using a microwave plasma generator. A vacuum chamber for CP-modified powder includes a microwave plasma generator, a glass dish holding the powder, a vacuum chamber, and a glass dish base in Figure 3. The h-BN nanoparticles were subjected to 10 min of CP modification. Table 2 shows the CP modification parameters.

#### 2.3.2. Preparation of h-BN Nanofluid

**Figure 4:**
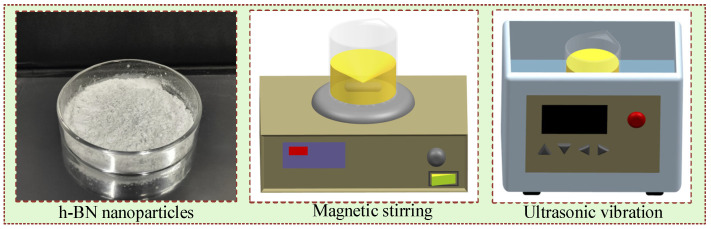
Preparation of h-BN nanofluids.

Three kinds of nanofluids were prepared using CO and h-BN nanoparticles. The first method was used to prepare surfactant-added nanofluids by mixing CO, unmodified h-BN nanoparticles, and APE-10. The second one was used to prepare plasma-modified nanofluid by mixing CO and CP (N_2_)-modified h-BN nanoparticles. The last one was used to prepare plasma-modified nanofluid by mixing CO and plasma (O_2_)-modified h-BN nanoparticles. The basic oil for h-BN nanofluid was CO, and the nanoparticle additive was made from h-BN nanoparticles, with a size of 50 nm. CO, 3 wt% surfactant (APE-10), and 0.3 wt% h-BN nanoparticles were stirred for 15 min, ultrasonicated for 60 min, and stirred for 30 min (Figure 4) [28,42,43].

#### 2.3.3. Friction and Wear Test

The friction wear experiments were conducted under the conditions of CO, h-BN nanofluid with added dispersant, and CP-modified nanofluid, respectively. To examine the stability of h-BN nanofluids, the h-BN nanofluids were produced and then subjected to friction and wear tests after 24 h of settling. The friction and wear tests were carried out using 7.5 N and 200 r/min, and friction and wear tests were conducted three times per operating condition with each test lasting 6 min. Therefore, as far as comparing the friction and wear performance of different lubricants was concerned, a one-factor test was used, where only the lubricant was changed, and other parameters were kept strictly the same.

## 3. Dispersion Mechanism

**Figure 5:**
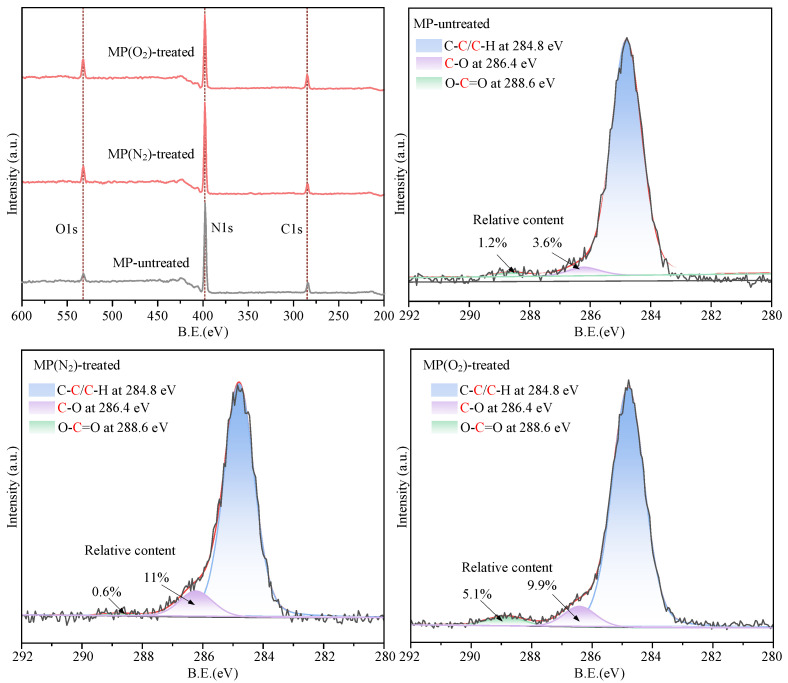
XPS analysis.

As shown in Figure 5, after CP processing, the relative content of O in h-BN nanoparticles increased, indicating the enhancement of oxygen-containing groups, and the rise in oxygen-containing polar groups enhanced the surface energy of h-BN nanoparticles. The increase in surface energy improves the surface wettability of h-BN nanoparticles. The increase in oxygen-containing groups is higher when the working gas is O_2_ than when the working gas is N_2_.

**Figure 6:**
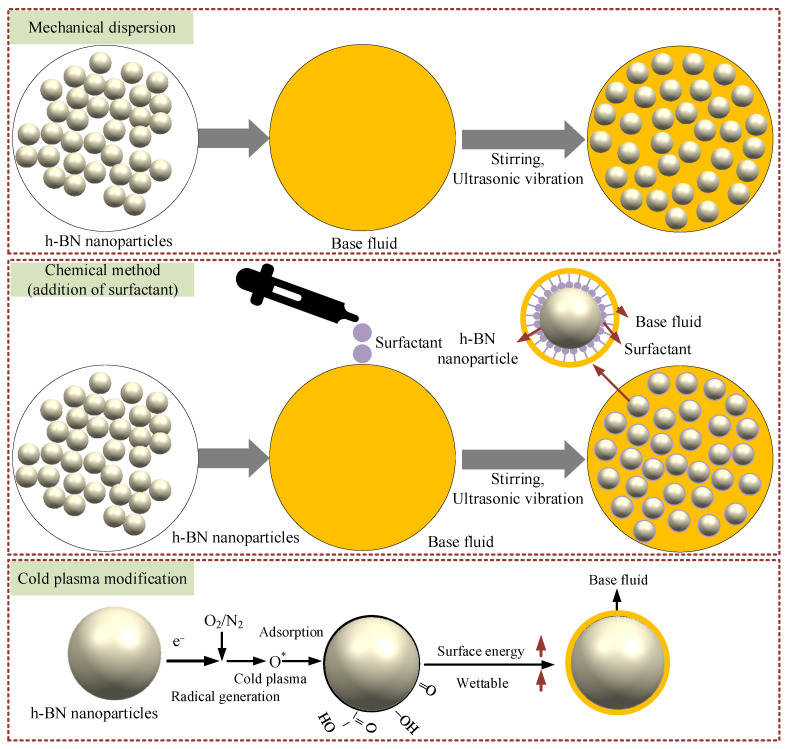
Dispersion mechanism.

The high-resolution C1s peaks on the surface of h-BN nanoparticles before and after treatment were mainly characterized as C-C/C-H, C-O, and O-C=O. As can be seen from the C1s peak, when the working gas is N_2_, the peak of O-C=O is significantly increased after CP treatment compared to the untreated surface of h-BN nanoparticles. When the working gas was O_2_, both C-O and O-C=O increased significantly. High-energy reactive ions bombard the surface of h-BN nanoparticles with inelastic collisions with the surface of h-BN nanoparticles. CP reactive particles such as O and -OH can break the nonpolar groups such as C-C and C-H on the molecular chains on the surface of the h-BN nanoparticles, forming dangling bonds and free radicals, and eventually generating oxygen-containing polar groups. The increase in the content of oxygen-containing polar groups increased the surface energy of h-BN nanoparticles. The surface wettability of h-BN nanoparticles was enhanced, and the h-BN nanoparticles were rapidly wetted and encapsulated by the base liquid and then form a stable dispersion (Figure 6). In the case of SNL, the base fluid mainly relies on hydrophobic–hydrophilic interactions to physically adsorb on the particle surface, forming a temporary protective layer, which is prone to desorption under high temperatures or shear stress [28].

## 4. Results and Discussion

### 4.1. Stability

**Figure 7:**
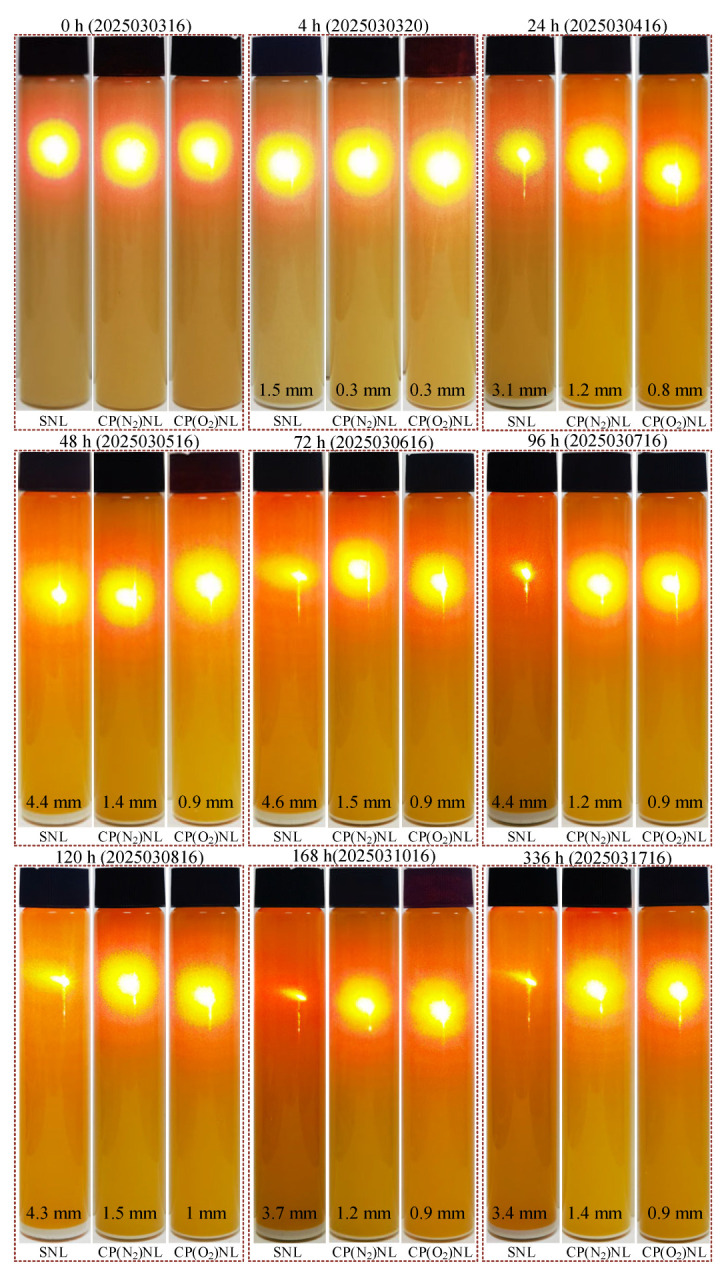
Observation of the dispersion stability of h-BN nanofluids.

After irradiating the prepared h-BN nanofluids with laser light, circular color scattering appeared, which was induced by the uniformly dispersed h-BN nanoparticles in the base fluid blocking the light source and scattering the light source to the surrounding area. The results indicate that at this time, the h-BN nanoparticles were well dispersed in the base fluid (Figure 7). However, after 4 h of static placement, the SNL showed significant sedimentation. By 24 h, the height of h-BN nanoparticles settling in SNL increased significantly. With laser irradiation, the circular dispersion of colors became significantly smaller compared to when it was just prepared. After 96 h of static placement, the h-BN nanoparticles in SNL almost completely settled. When the laser irradiated the reagent vial, the laser penetrated the vial, indicating that the nanoparticles at the top of the vial were almost completely agglomerated and settled to the bottom.

The increase in the surface energy of h-BN nanoparticles after CP treatment can enhance the wettability of the liquid on its surface, which in turn improves the compatibility of the base liquid with the nanoparticles. In the CP-modified h-BN nanofluid, which was stationary for 4 h, a very small amount of nanoparticles precipitated at the bottom of the reagent vial. After 96 h of settling, the agglomeration and settling of h-BN nanoparticles on the bottom increased significantly compared to 4 h. Irradiation of the reagent vial tip using laser light did not penetrate the vial, demonstrating that the tip was still uniformly dispersed with h-BN nanoparticles. Continued static placement for 120 h, 168 h, and 336 h revealed that the h-BN nanoparticles settling at the bottom of the reagent vials were not significantly different from those at 96 h. However, the CP-modified h-BN nano-sedimentation produced using N_2_ as the working gas was slightly higher than that of O_2_. As revealed by previous XPS analysis, the CP generated with O_2_ as the working gas is better than N_2_ for the surface modification of nanoparticles. From the static observation, the amount of agglomerate deposition of CP-modified h-BN nanoparticles is much less than that of SNL. The dispersion stability exceeds 336 h, indicating that the CP-modified h-BN nanoparticles have better suspension stability in oil.

### 4.2. Kinematic Viscosity

**Figure 8:**
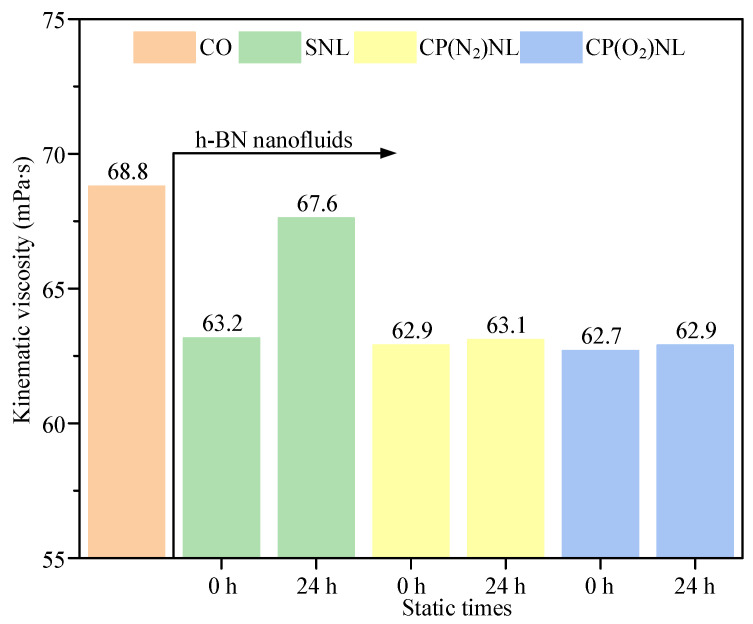
h-BN nanofluid viscosity.

The kinematic viscosity of nanofluids is measured using a cone-plate rheometer. The cone-plate rheometer consists mainly of a horizontal plate and an interchangeable conical rotor. The motor drives the conical rotor to rotate at a constant speed. An appropriate amount of fluid is kept in the angle between the horizontal plate and the conical rotor, and the friction of the fluid drives the conical plate to rotate. Under the action of the torque sensor, the conical rotor rotates at a certain angle and then reaches equilibrium. At this point, the applied torque is associated with the friction of the fluid being measured: the greater the fluid viscosity is, the greater the torque. The testing principle of the cone-plate rheometer is therefore completely different from that of the numerical rotational viscometer. The numerical rotational viscometer utilizes the viscosity of the liquid. The cone-plate rheometer utilizes the friction between the horizontal plate and the conical rotor. The viscosity of CO previously tested by researchers using a numerical rotational viscometer was 49–51 mPa∙s, and the viscosity increased slightly with increasing nanoparticle concentration [44]. The viscosity of CO tested using the cone-plate rheometer was 68.8 mPa∙s. However, the introduction of h-BN nanoparticles reduced the tested viscosity instead, which was contrary to the results of the rotational viscometer test because it could act as a friction reducer and exhibited anti-wear properties so that the friction between the horizontal plate and the conical rotor decreased. The kinematic viscosity values tested using the cone-plate rheometer are averages calculated by the system. The nanofluid with added h-BN nanoparticles has better lubrication properties than the base fluid, and the friction between the horizontal plate and the conical rotor was reduced. Thus, the measured kinematic viscosity value was smaller, roughly around 63 mPa∙s, which was 8.4% lower than that of the base fluid 68.8 mPa∙s in Figure 8. For the surfactant-added h-BN nanofluid, the kinematic viscosity increased from 63.2 mPa∙s to 67.6 mPa∙s after 24 h of settling, indicating that a large amount of nanoparticle sedimentation occurred. The CP-modified h-BN nanofluids showed less change 24 h after preparation, with the lowest value of CP(O_2_)NL being the most effective. Compared to N_2_, after the CP generated using O_2_ as the working gas to treat the nanoparticles, the surface of the nanoparticles had relatively high oxygen-containing polar groups, and the surface wettability was better. Thus, the base solution was more likely to encapsulate the nanoparticles to reduce the agglomeration between the particles.

### 4.3. Heat Transfer Performance

**Figure 9:**
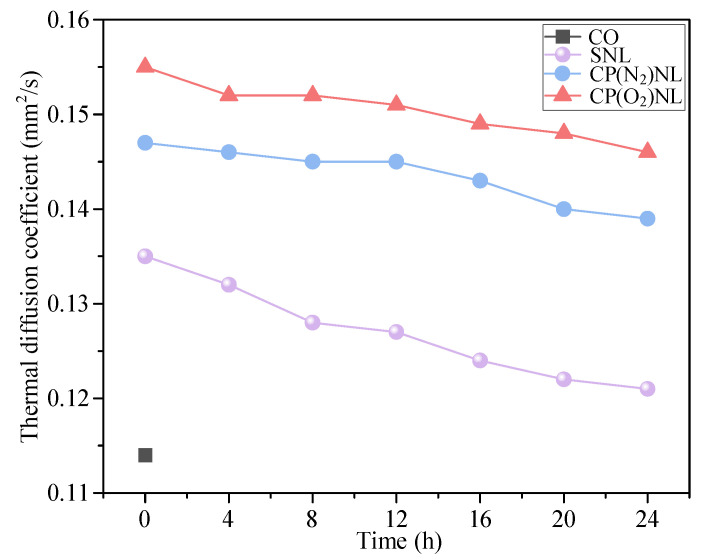
Thermal diffusion coefficients of different lubricants.

The thermal diffusion coefficient describes the ability to conduct heat inside the nanofluid. The thermal diffusion coefficient of the base liquid CO was minimized to 0.117 mm^2^/s (Figure 9). The thermal diffusion coefficients of SNL, CP(N_2_)NL, and CP(O_2_)NL increased after the addition of the h-BN nanoparticles to 0.135, 0.147, and 0.155 mm^2^/s, respectively, but that of the SNL decreased dramatically after 24 h of static placement to 0.121 mm^2^/s. For the CP-modified h-BN nanofluids, the thermal diffusion coefficients were reduced to 0.139 and 0.146, indicating that they were uniformly dispersed and had better stability. Nanoparticles have a large specific surface area and a large contact area with fluid molecules. Since the heat transfer performance of nanoparticles is usually higher than those of conventional fluids, when the particles collide with the fluid molecules, the heat can be transferred more efficiently from the high-temperature region to the low-temperature region, thus enhancing the heat transfer. Therefore, the thermal diffusion coefficient of the nanofluid is affected by the nanoparticle content. When many h-BN nanoparticles undergo settling, the concentration of nanoparticles in the h-BN nanofluid decreases, further reducing the thermal diffusion coefficient.

### 4.4. Tribological Performance of h-BN Nanofluids

#### 4.4.1. Coefficient of Friction

**Figure 10:**
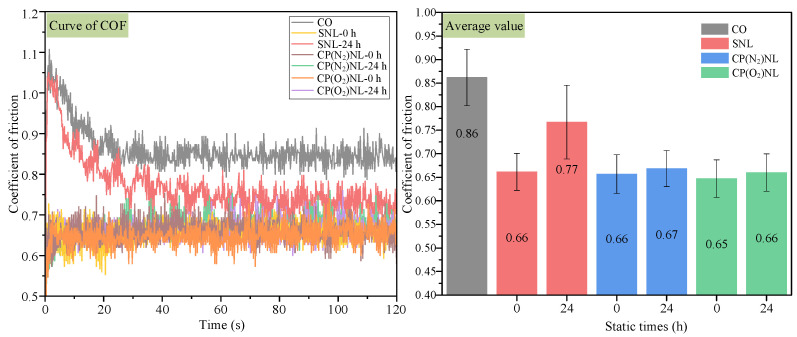
Coefficient of friction of lubricants.

The coefficient of friction can be used as an important basis for evaluating the performance of cooling and lubricating mediums. By experimentally measuring the coefficient of friction of different lubricants under specific conditions, it is possible to compare their cooling and lubricating effects and thus select a medium with better performance [45,46,47]. As shown in Figure 10, the coefficient of friction curve is higher than other conditions when cotton oil is added to the friction wear area, and its average value is 0.86, reflecting a poor friction environment. The coefficient of friction curves for the SNL and CPNL tests are significantly lower than CO, and all have a mean value of about 0.66, which is 23.3% lower than the dry condition. When the SNL was placed statically for 24 h, the coefficient of friction was raised to 0.77 compared with that of the freshly prepared SNL, indicating that the nanoparticles in the SNL had undergone sedimentation. CP(N_2_)NL and CP(O_2_)NL showed slight fluctuations in the friction coefficients tested after 24 h of static placement, indicating that the CP-modified h-BN nanoparticles were better dispersed and stabilized in CO. After static placement of CP-modified h-BN nanofluid, the amount of nanoparticles settling is low, and the nanofluid contains sufficient nanoparticles to form a stable oil film between the friction vice. In addition, the results obtained for this h-BN nanofluid coincide with the results of Yildirim et al. that indicate that the nanoparticles roll freely between the friction partners, converting sliding friction into rolling friction and significantly reducing the coefficient of friction [18].

#### 4.4.2. Three-Dimensional Surface Topography of the Sample Disk

**Figure 11:**
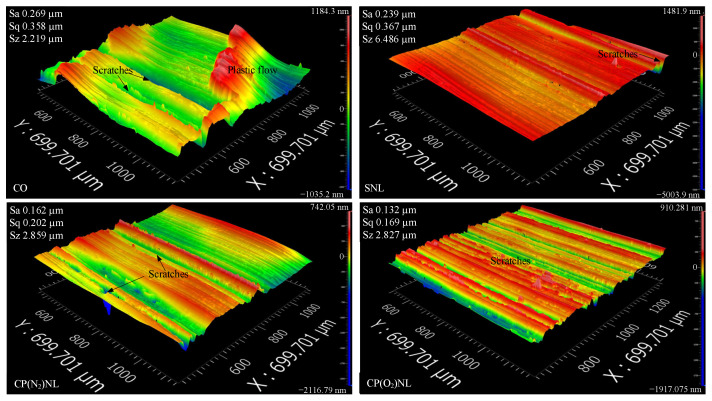
Three-dimensional surface morphology of friction samples.

Figure 11 presents the three-dimensional morphological characteristics of the wear samples under various conditions. The three-dimensional surface morphology under each condition was measured randomly six times. Under the CO condition, the surface of the friction disk showed plastic material flow. Under the SNL condition, the surface is flatter, but there are large depth scratches. Under the CP(N_2_)NL and CP(O_2_)NL conditions, there is not much difference between the two surfaces, and both of them have more scratches, but with less depth.

#### 4.4.3. Surface Roughness of the Friction Disc

**Figure 12:**
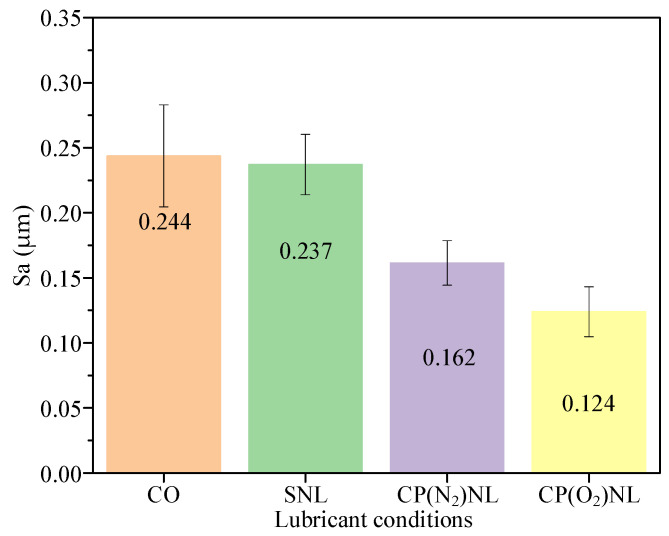
Surface roughness of friction samples.

Surface roughness quantifies the quality of the workpiece surface [48,49]. After conducting friction and wear experiments under different conditions, six areas were randomly taken from the surface of the friction discs to measure the surface roughness Sa and to obtain the mean value (Figure 12). The Sa value of the friction discs under the CO condition is 0.244 µm, which is due to the lack of lubricant in the friction area. In addition, the aluminum alloy is a plastic material, which leads to adhesion and spalling of the material. After 24 h of settling, the Sa value of the sample disc under the SNL condition was 0.237 µm, while that of the friction specimen under the CP(N_2_)NL condition was 0.162 µm, indicating that CP(N_2_)NL can effectively enhance the suspension stability of h-BN nanoparticles. The surface roughness is minimized under the CP(O_2_)NL conditions, corresponding to a somewhat smoother 3D surface topography, indicating that the dispersion stability of CP(O_2_)NL is superior to that of CP(N_2_)NL. Table 3 shows the comparison of the performance of different nanofluids.

#### 4.4.4. Scratch Depth

**Figure 13:**
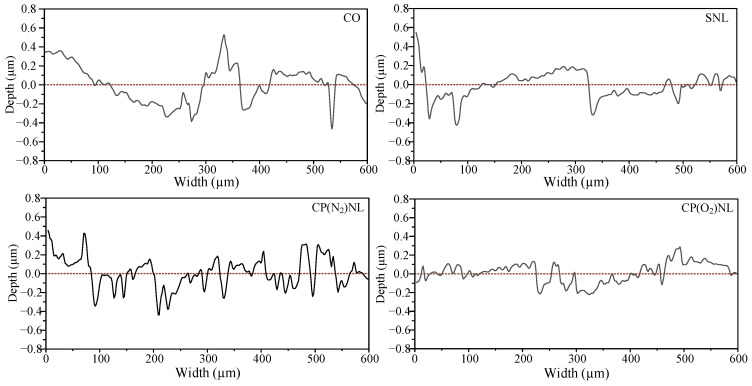
Scratch depth under different lubricant conditions.

The depth of the scratches in Figure 12 was randomly measured based on the mean value of Sa at each operating condition and the area closest to the mean value among the six areas. The maximum depth of scratches on friction disc surface was higher than 0.4 µm, and the scratches were irregular under both CO and SNL (after 24 h of settling) conditions (Figure 13). For the CP-modified h-BN nanofluid, the scratch depth on the surface of the friction samples is relatively small, and the fluctuation of the scratches is regular, which indicates that the lubrication environment is relatively good when the friction wear experiments are conducted.

#### 4.4.5. Micro-Morphology

**Figure 14:**
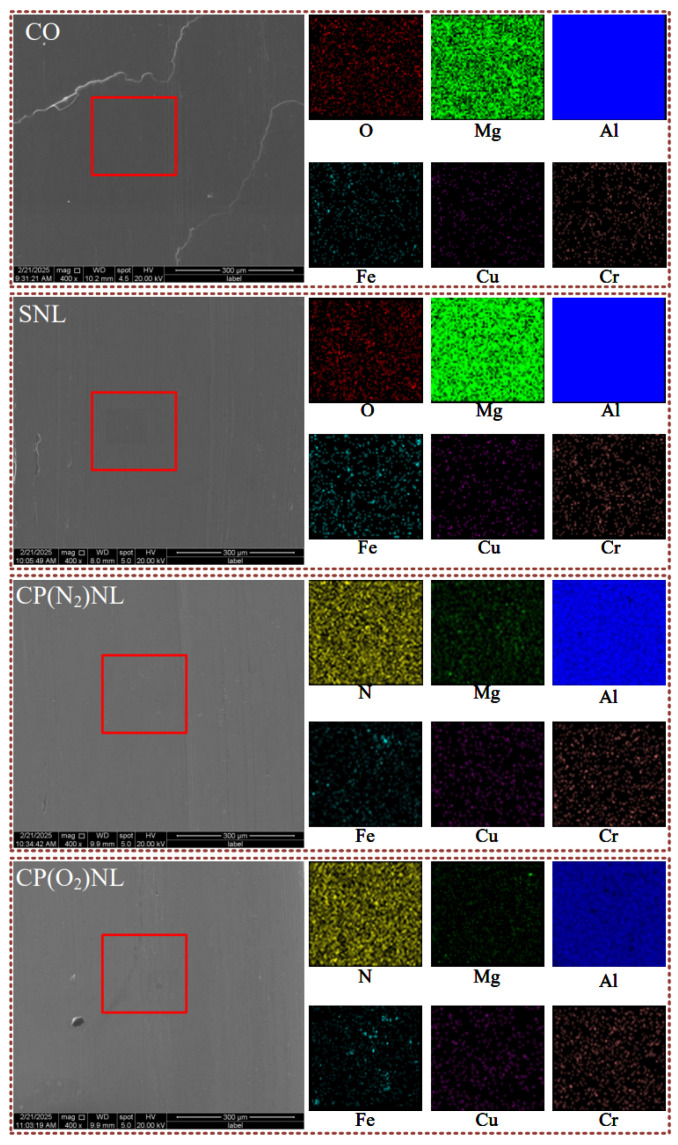
Surface morphology of friction wear samples.

The micro-morphology of the friction sample can also qualitatively reflect the surface characteristics of the friction sample [50,51,52,53]. The surface material of the friction wear samples plastically slipped under CO conditions (Figure 14). Elemental analysis of the surface of the friction disc shows that more oxygen elements appeared on the surface of the friction disc under CO and SNL conditions, which may be the result of a small amount of oxidative wear that may have occurred on the surface of the friction wear samples due to the friction of the pins and discs in the friction process. Under other conditions, no oxygen elements were found to be particularly noticeable. SNL, CP(N_2_)NL, and CP(O_2_)NL were subjected to friction wear experiments after 24 h of settling, and the surface scratches and flaking were more pronounced on the friction samples under SNL conditions. Plastic slip of the material under CO conditions and surface scratches and flaking under SNL conditions can lead to surface bumps and depressions and hence higher Sa values. In addition, the surface of the CP-modified nanofluid counterpart sample is relatively smooth, which is due to the better suspension stability of the CP-modified nanoparticles. Therefore, the CP-modified nanofluid corresponds to a relatively low Sa value of the friction surface.

## 5. Conclusions

In this research, we evaluated the dispersion mechanism of SNL, CP(N_2_)NL, and CP(O_2_)NL prepared using different methods with XPS. The dispersion stability of h-BN nanofluids was evaluated using static observation. Finally, friction and wear experiments were carried out under different lubrication conditions, and the coefficient of friction, three-dimensional surface morphology, Sa value, surface scratches, microscopic morphology, and heat transfer properties were analyzed under various lubrication conditions. The conclusions are as follows:

(1) The surface of h-BN nanoparticles after CP treatment shows an increase in polar groups and surface energy, improving the wettability of the base solution on the nanoparticles. Among them, the plasma modification using O_2_ as the working gas had the best effect.
(2) Among SNL, CP(N_2_)NL, and CP(O_2_)NL, the nanofluid, CP(O_2_)NL, prepared using CP(O_2_)-modified h-BN, is stable for more than 336 h. It has excellent dispersion stability with a viscosity of 62.9 mPa∙s and a thermal diffusion coefficient of 0.155 mm^2^/s.
(3) For the friction wear test, the coefficient of friction under the cotton seed oil condition was 0.86, and the Sa value of the disc was 0.244 µm, with a scratch depth higher than 0.4 µm. The tribological performance of SNL was not much different from that of CO after static placement for 24 h. In contrast, the nanofluids prepared from CP-modified h-BN have a friction coefficient of about 0.66 before and after 24 h of static observation, and the Sa values of the discs are 31.6–49.2% lower than those of the CO.

## Figures and Tables

**Table 1:** Chemical composition of aluminum alloy 6061-T651.

Element	Si	Fe	Cu	Mn	Mg	Cr	Zn	Ti	Al
Measured value	0.66	0.4	0.27	0.12	1.1	0.33	0.03	0.03	Remainder

**Table 2:** Parameters of CP modification.

Parameter	Value
Power frequency of CP (MHz)	2450
Power of CP (W)	150
O_2_/N_2_ flow of CP (mL/min)	80
CP treatment time (min)	10

**Table 3:** Stability performance of different lubricants.

Lubricant	Kinematic Viscosity (mPa∙s)	Thermal Diffusion Coefficient (mm^2^/s)	Sa (µm)
CO	68.8	0.117	0.244
SNL	63.2–67.6 (0–24 h)	0.135–121 (0–24 h)	0.237
CO(N_2_)NL	62.9–63.1 (0–24 h)	0.147–0.139 (0–24 h)	0.162
CO(O_2_)NL	62.7–62.9 (0–24 h)	0.155–0.146 (0–24 h)	0.124

## Data Availability

Data are contained within the article.

## Abbreviations

The following abbreviations are used in this manuscript:

SNL	h-BN nanofluid with added surfactant
CP(N2)NL	Cold plasma-modified h-BN nanofluid with N2 as the working gas
CP(O2)NL	Cold plasma-modified h-BN nanofluid with O2 as the working gas
CP	Cold plasma
XPS	X-ray photoelectron spectroscopy
Sa	Surface roughness
CO	Cottonseed oil
PVP	Polyvinylpyrrolidone
CTAB	Cetyltrimethylammonium bromide
GA	Gum arabic
SDS	Sodium dodecyl sulfate
SDOC	Sodium deoxycholate
SLS	Sodium dodecyl sulfate
MP	Microwave plasma

## References

[1] Akram S., Athar M., Saeed K., Razia A., Alghamdi M., Muhammad T. (2022). Impact of Partial Slip on Double Diffusion Convection of Sisko Nanofluids in Asymmetric Channel with Peristaltic Propulsion and Inclined Magnetic Field. Nanomaterials.

[2] Wohld J., Beck J., Inman K., Palmer M., Cummings M., Fulmer R., Vafaei S. (2022). Hybrid Nanofluid Thermal Conductivity and Optimization: Original Approach and Background. Nanomaterials.

[3] Shi R., Lin J., Yang H. (2022). Particle Distribution and Heat Transfer of SiO_2_/Water Nanofluid in the Turbulent Tube Flow. Nanomaterials.

[4] Yang M., Ma H., Hao J.C., Li Z.H., Li R.Z., Zhou Z.M., Gao T., Liu M.Z., Cui X., Wang X.M. (2024). Droplet size distribution model of needle electrode electrostatic atomization and milling nickel-based alloy performance evaluation. J. Manuf. Process..

[5] Mousavi S.B., Pourpasha H., Heris S.Z. (2024). High-temperature lubricity and physicochemical behaviors of synthesized Cu/TiO_2_/MnO_2_-doped GO nanocomposite in high-viscosity index synthetic biodegradable PAO oil. Int. Commun. Heat Mass Transf..

[6] Mousavi S.B., Heris S.Z., Estellé P. (2020). Experimental comparison between ZnO and MoS_2_ nanoparticles as additives on performance of diesel oil-based nano lubricant. Sci. Rep..

[7] Mousavi S.B., Heris S.Z. (2020). Experimental investigation of ZnO nanoparticles effects on thermophysical and tribological properties of diesel oil. Int. J. Hydrogen Energy.

[8] Duan Z., Xi S., Wang S., Wang Z., Bian P., Li C., Song J., Liu X. (2024). Identification of Cutting Workpiece Surface Defects Based on an Improved Single Shot Multibox Detector. Intell. Sustain. Manuf..

[9] Mousavi S.B., Heris S.Z., Hosseini M.G. (2019). Experimental investigation of MoS_2_/diesel oil nanofluid thermophysical and rheological properties. Int. Commun. Heat Mass Transf..

[10] Gong P., Zhang Y.B., Wang C.J., Cui X., Li R.Z., Sharma S., Liu M.Z., Gao T., Zhou Z.M., Wang X.M. (2024). Residual stress generation in grinding: Mechanism and modeling. J. Mater. Process. Tech..

[11] Yang M., Kong M., Li C.H., Long Y.Z., Zhang Y.B., Sharma S., Li R.Z., Gao T., Liu M.Z., Cui X. (2023). Temperature field model in surface grinding: A comparative assessment. Int. J. Extreme. Manuf..

[12] Duan Z.J., Wang Z.H., Wang S.S., Zhang B.Z., Bian Peng B., Li Y.H., Liu J.Y., Song J.L., Li C.H., Liu X. (2025). Tool wear in enhanced minimum quantity lubrication assisted milling: From mechanism to application. Chin. J. Aeronaut..

[13] Gao T., Xu P.M., Wang W., Zhang Y.B., Xu W.H., Wang Y.Q., An Q.L., Li C.H. (2024). Force model of ultrasonic empowered minimum quantity lubrication grinding CFRP. Int. J. Mech. Sci..

[14] Anand N., Kumar A.S., Paul S. (2019). Effect of cutting fluids applied in MQCL mode on machinability of Ti-6Al-4V. J. Manuf. Process..

[15] Talib N., Rahim E.A. (2018). Performance of modified jatropha oil in combination with hexagonal boron nitride particles as a bio-based lubricant for green machining. Tribol. Int..

[16] Boukheit A., Chabert F., Otazaghine B., Taguet A. (2022). h-BN Modification Using Several Hydroxylation and Grafting Methods and Their Incorporation into a PMMA/PA6 Polymer Blend. Nanomaterials.

[17] Yun H., Kwak M.-G., Park K., Kim Y. (2022). Fabrication, Thermal Conductivity, and Mechanical Properties of Hexagonal-Boron-Nitride-Pattern-Embedded Aluminum Oxide Composites. Nanomaterials.

[18] Yildirim C.V. (2019). Experimental comparison of the performance of nanofluids, cryogenic and hybrid cooling in turning of Inconel 625. Tribol. Int..

[19] Said Z., Sundar L.S., Tiwari A.K., Ali H.M., Sheikholeslami M., Bellos E., Babar H. (2022). Recent advances on the fundamental physical phenomena behind stability, dynamic motion, thermophysical properties, heat transport, applications, and challenges of nanofluids. Phys. Rep..

[20] Bakthavatchalam B., Habib K., Saidur R., Saha B.B., Irshad K. (2020). Comprehensive study on nanofluid and ionanofluid for heat transfer enhancement: A review on current and future perspective. J. Mol. Liq..

[21] Xia G.D., Jiang H.M., Liu R., Zhai Y.L. (2014). Effects of surfactant on the stability and thermal conductivity of Al_2_O_3_/de-ionized water nanofluids. Int. J. Therm. Sci..

[22] Altun A., Sara O.N., Simsek B. (2021). A comprehensive statistical approach for determining the effect of two non-ionic surfactants on thermal conductivity and density of Al_2_O_3_-water-based nanofluids. Colloids Surf. A.

[23] Mostafizur R.M., Rasul M.G., Nabi M.N. (2022). Effect of surfactant on stability, thermal conductivity, and viscosity of aluminium oxide-methanol nanofluids for heat transfer applications. Therm. Sci. Eng. Prog..

[24] Sezer N., Koç M. (2018). Stabilization of the aqueous dispersion of carbon nanotubes using different approaches. Therm. Sci. Eng. Prog..

[25] Almanassra I.W., Manasrah A.D., Al-Mubaiyedh U.A., Al-Ansari T., Malaibari Z.O., Atieh M.A. (2020). An experimental study on stability and thermal conductivity of water/CNTs nanofluids using different surfactants: A comparison study. J. Mol. Liq..

[26] Ebrahim S.A., Pradeep E., Mukherjee S., Ali N. (2023). Rheological behavior of dilute graphene-water nanofluids using various surfactants: An experimental evaluation. J. Mol. Liq..

[27] Ibrahim A.M.M., Li W., Zeng Z.X., Bedairi B.H., Elsheikh A. (2025). Graphene nanoplatelets-water nanofluids: A sustainable approach to enhancing Ti-6Al-4V grinding performance through minimum quantity lubrication. Tribol. Int..

[28] Gao T., Li C.H., Zhang Y.B., Yang M., Jia D.Z., Jin T., Hou Y.L., Li R.Z. (2019). Dispersing mechanism and tribological performance of vegetable oil-based CNT nanofluids with different surfactants. Tribol. Int..

[29] Musavi S.H., Davoodi B., Niknam S.A. (2019). Effects of reinforced nanoparticles with surfactant on surface quality and chip formation morphology in MQL-turning of superalloys. J. Manuf. Process..

[30] Sirin E., Kivak T., Yildirim Ç.V. (2021). Effects of mono/hybrid nanofluid strategies and surfactants on machining performance in the drilling of Hastelloy X. Tribol. Int..

[31] Wang R.T., Yang C.A., Chen S.L., Chang T.L., Wang J.C. (2025). Investigations of thermoelectric properties in polymer-based hybrid nanofluids with various surfactants. J. Phys. Chem. Solids.

[32] Yang B., Shi Y., Ma X.Q., Yu X.H. (2023). Effects of mixed anionic/cationic surfactants on ZnO nanofluid. J. Mol. Liq..

[33] Krishnamurthy A., Kaladgi A.R., Halemani B.S., Buradi A., Afzal A., Saleel C.A. (2021). Performance enhancement in tribological properties of lubricants by dispersing TiO_2_ nanoparticles. Mater. Today Proc..

[34] Liu X., Wang B.Q., Li Y.H., Zhou Y.Y., Zhang J.H., Wang Z.H., Yan J.C., Gu X.L., Yuan Z.Z., Chen Y. (2023). Improving machinability of single-crystal silicon by cold plasma jet. J. Manuf. Process..

[35] Wang Z.H., Duan Z.J., Wang S.S., Li Y.H., Zhou Y.Y., Liu J.Y., Liu X. (2025). Influence of cold plasma on material removal behavior during diamond grit scratching single crystal silicon. Colloid Surf. A.

[36] Wang Z., Yang W., Duan Z., Wang S., Li Y.-h., Zhou Y., Liu J., Song J., Liu X. (2024). Experimental Study on Cold Plasma Jet (CPJ) Assisted Micro-Milling of 30CrMnSiNi2A. Intell. Sustain. Manuf..

[37] Wang Z.H., Li Y.H., Wang S.S., Duan Z.J., Cao X.M., Zhou Y.Y., Liu X., Liu J.Y. (2024). Feasibility and mechanism of atmospheric pressure cold plasma jet (APCPJ) assisted micro-milling of bulk metallic glasses (BMGs). Ceram. Int..

[38] Liu J.Y., Wang S.S., Li Y.H., Duan Z.J., Ning L.J., Wang Z.H., Chen Y., Liu X. (2024). High-efficiency and low-damage modification of engineering metal materials by oxygen-mixing atmospheric pressure cold plasma jets. Appl. Surf. Sci..

[39] Duan Z.J., Wang S.S., Li C.H., Wang Z.H., Bian P., Sun J., Song J.L., Liu X. (2024). Cold plasma and different nano-lubricants multi-energy field coupling-assisted micro-milling of Al-Li alloy 2195-T8 and flow rate optimization. J. Manuf. Process..

[40] Duan Z.J., Wang S.S., Wang Z.H., Li C.H., Li Y.H., Song J.L., Liu J.Y., Liu X. (2024). Tool wear mechanisms in cold plasma and nano-lubricant multi-energy field coupled micro-milling of Al-Li alloy. Tribol. Int..

[41] Put S., Bertels C., Vanhulsel A. (2013). Atmospheric pressure plasma treatment of polymeric powders. Surf. Coat. Tech..

[42] Yildirim C.V., Sarikaya M., Kivak T., Sirin S. (2019). The effect of addition of hBN nanoparticles to nanofluid-MQL on tool wear patterns, tool life, roughness and temperature in turning of Ni-based Inconel 625. Tribol. Int..

[43] Bian P., Duan Z.J., Jia Y.S., Wang Z.H., Wang S.S., Tan J., Zhou Y.Y., Song J.L., Liu X. (2025). Evaluation of Surface Integrity of Multi-Energy Field Coupling-Assisted Micro-Grinding Hastelloy Alloy. Micromachines.

[44] Duan Z.J., Wang S.S., Li C.H., Wang Z.H., Bian P., Song J.L., Liu X. (2025). Tribological and micro-milling performance of surfactant-free microwave plasma-modified Al_2_O_3_ nanoparticles biodegradable lubricants. J. Clean. Prod..

[45] Yang M., Hao J.C., Wu W.T., Li Z.H., Ma Y.Q., Zhou Z.M., Gao T., Liu M.Z., Cui X., Zhang Y.B. (2024). Critical cutting thickness model considering subsurface damage of zirconia grinding and friction—wear performance evaluation applied in simulated oral environment. Tribol. Int..

[46] Gao T., Liu J.X., Sun X.F., Zhang Y.B., Yang M., Liu M.Z., Xu W.H., An Q.L., Wang D.Z., Xu P.M. (2025). Enhanced permeation mechanism and tribological assessment of ultrasonic vibration nanolubricants grinding CFRP. Tribol. Int..

[47] Mousavi S.B., Heris S.Z., Estellé P. (2021). Viscosity, tribological and physicochemical features of ZnO and MoS_2_ diesel oil-based nanofluids: An experimental study. Fuel.

[48] Kai X.U., Yun Y., Wei F., Min W.A.N., Weihong Z. (2024). Internal cooling techniques in cutting process: A review. J. Adv. Manuf. Sci. Technol..

[49] Danni L.U., Kaining S.H.I., Jiale L.I., Huhu L.I., Yuchang F.A.N., Zhen C., Yaoyao S.H.I. (2024). Surface generation mechanism and efficiency improvement in ultrasonic vibration assisted belt flapwheel flexible polishing GH4169. J. Adv. Manuf. Sci. Technol..

[50] Sun Y., Sun Y.R., Huang Y.M., Gong S.Q., Sun M.S., Liu M. (2025). Study on developing predicted system model of cutting-edge trajectory for micro-milling process based on tool runout error, chip thickness and force signal. Mech. Syst. Signal Process..

[51] Qu S.S., Wei C.X., Yang Y.Y., Yao P., Chu D.K., Gong Y.D., Zhao D., Zhang X.P. (2024). Grinding mechanism and surface quality evaluation strategy of single crystal 4H-SiC. Tribol. Int..

[52] Zhao G.L., Zhao B., Ding W.F., Xin L.J., Nian Z.W., Peng J.H., He N., Xu J.H. (2024). Nontraditional energy-assisted mechanical machining of difficult-to-cut materials and components in aerospace community: A comparative analysis. Int. J. Extreme. Manuf..

[53] Xu Q.H., Wang J.L., Wang Y.Q., Gao H. (2025). Exploring the anisotropic damage behaviour during the scratching process of SiCf/SiC composites. Compos. Part A Appl. Sci. Manuf..

